# Light-Chain Amyloidosis With Peripheral Neuropathy as an Initial Presentation

**DOI:** 10.3389/fneur.2021.707134

**Published:** 2021-09-28

**Authors:** Min Qian, Lan Qin, Kaini Shen, Hongzhi Guan, Haitao Ren, Yanhuan Zhao, Yuzhou Guan, Daobin Zhou, Bin Peng, Jian Li, Lin Chen

**Affiliations:** ^1^Department of Neurology, Peking Union Medical College Hospital, Chinese Academy of Medical Sciences Beijing, Beijing, China; ^2^Department of Neurology, University of Massachusetts Medical School, Worcester, MA, United States; ^3^Department of Hematology, Peking Union Medical College Hospital, Chinese Academy of Medical Sciences Beijing, Beijing, China

**Keywords:** primary light chain amyloidosis, peripheral neuropathy, chemotherapy, outcome, nerve biopsy

## Abstract

**Objective:** This study aimed to better understand the clinical, electrophysiological, pathological features and prognosis of peripheral nerve involvements in primary immunoglobulin light-chain (AL) amyloidosis.

**Methods:** We retrospectively reviewed the clinical data of eight AL amyloidosis patients with peripheral neuropathy as the initial presentation including clinical features, histopathological findings and treatment.

**Results:** There were seven males and one female aged from 52 to 66 years. Initial symptoms included symmetrical lower extremity numbness, lower extremity pain and carpal tunnel syndrome. Seven patients suffered from severe pain and required pain management. Six patients had predominant autonomic dysfunction. Six patients had cardiac involvement, and one patient had renal involvement. Monoclonal proteins were found in all patients, with IgA λ in one, IgG λ in two, λ alone in three, κ alone in one and IgM κ in one. Sural nerve biopsies were performed in 7 cases, all of which showed amyloid deposition in the endoneurium (in the perivascular region in some cases), in addition to moderate to severe myelinated fiber loss with axonal degeneration. Six patients were treated with combined chemotherapy. In three patients who began chemotherapy earlier (6–10 months after onset), two achieved a hematological complete response, and one achieved a partial response. three patients who had delayed chemotherapy (36 months after onset) died between 5 and 12 months after diagnosis.

**Conclusion:** Early recognition of AL amyloidosis with peripheral neuropathy as the initial symptom is very important. Nerve biopsy can help to make the diagnosis. Early diagnosis and chemotherapy are critical to achieve better outcomes.

## Introduction

Peripheral neuropathy is a common neurological disorder with different etiologies (1). Amyloidosis is a rare cause of peripheral neuropathy and is often overlooked (2). Depending on the type of precursor protein, amyloidosis is usually divided into various types, including immunoglobulin amyloid light chain (AL), amyloid A (AA), and amyloid transthyretin (ATTR). AL is the most common form of amyloidosis (2). AL amyloidosis results from the overproduction of monoclonal immunoglobulin light chains and the subsequent deposition of light chains or their variable components. This insoluble protein deposits in tissues and interferes with organ function, including the heart, kidneys, liver, gastrointestinal tract and peripheral nerves (2).

Peripheral neuropathy is a common manifestation of AL amyloidosis, and the incidence of peripheral neuropathy in AL amyloidosis varies from 9.6 to 35% (3–5). The typical pattern of amyloid neuropathy is symmetrical, length-dependent, lower-limb predominant, slowly progressing polyneuropathy, with severe pain. The involvement of small nerve fibers is more severe than that of large nerve fibers. Over time, both motor and sensory fibers and both large and small fibers become involved (5). Autonomic neuropathy is a particularly severe complication that manifests with gastroparesis, diarrhea or constipation, impotence and severe postural hypotension (6). Carpal tunnel syndrome is also common in AL amyloidosis (6).

Peripheral nerve involvement in AL amyloidosis is not well recognized due to a lack of awareness. When the initial symptoms present as peripheral neuropathy, there is often a delay in the diagnosis (3, 7). In this study, we retrospectively studied peripheral nerve involvement in AL amyloidosis patients with regard to clinical manifestations, electrophysiological characteristics, pathological changes and treatments.

## Patients and Methods

The clinical data of eight patients with AL amyloidosis with peripheral neuropathy referred to the Neurology Department of Peking Union Medical College Hospital between 2010 and 2019 were retrospectively reviewed. All patients had the following characteristics: 1. Clinical manifestations, signs and electrophysiological changes of peripheral neuropathy; 2. nerve biopsy or muscle biopsy (polarized microscopy and fluorescence microscopy) showing amyloid deposition; 3. no family history; 4. blood/urine immunofixation electrophoresis/free light chain confirming the presence of monoclonal protein; 5. bone marrow aspiration or biopsy and systemic evaluation excluding other plasma cell diseases, such as macroglobulinemia, multiple myeloma or POEMS; and 6. other etiologies of peripheral neuropathy were excluded, including diabetic,autoimmune disease, paraneoplastic, inflammatory, alcohol, hereditary and so on. The study was approved by Ethics Review Committee of Peking Union Medical College Hospital, Chinese Academy of Medical Sciences. We received the written informed patient consent from all the patients.

A comprehensive baseline evaluation of the patients was performed, including a systemic examination and examinations of liver and kidney function, serum protein electrophoresis, serum immunofixation electrophoresis, urine immunofixation electrophoresis, serum free light chain (FLC), cTnI, and NT-proBNP or BNP. The presence of visceral organ involvement was assessed in each patient based on physical examinations and laboratory data (8). Three patients underwent brain MRI scans. Routine sensory and motor conduction study, needle electrography, F waves and skin sympathetic reflex (SSR) were performed on all the patients. All of the patients were followed up regularly.

Sural nerve biopsy was performed in seven patients. A nerve fragment was fixed in 10% buffered formalin, embedded in paraffin, and stained with hematoxylin and eosin, Masson's trichrome and Congo red. A second nerve fragment was fixed by immersion in 2.5% buffered glutaraldehyde, postfixed in 1% osmium tetroxide, and prepared in the usual manner in semithin sections for electron microscopy. The nerve fragments were fixed in glutaraldehyde and stained with osmic acid. In one patient, specimens from the quadriceps muscle were taken and prepared into cryosections. Congo red staining were performed. Rectal mucosa biopsy, abdominal wall fat pad biopsy and gingiva biopsy were performed in four cases. Hematoxylin and eosin and Congo red staining were performed.

## Results

### Clinical Characteristics

The clinical profiles are summarized in Table 1. There were seven males and one female, and the median age was 61 (range, 52–66) years. Initial symptoms included symmetrical lower extremity numbness in three patients and lower extremity pain in four patients. Seven patients suffered from severe neuropathic pain and required pain management. Five patients had autonomic involvement, including gastrointestinal symptoms (alternative diarrhea or constipation), sexual dysfunction, orthostatic hypotension, urinary dysfunction, and decreased sweating. One patient had orthostatic hypotension, urine dysfunction, and reduced sweating. Five patients complained of limb weakness with distal predominance. Patient 6 complained of weakness of the proximal lower limb. Patient 6 also presented with numbness in both hands with electrophysiology evidence of carpal tunnel syndrome and weakness of the proximal lower limbs with EMG evidence of myopathy. All patients had significant weight loss. Six patients had cardiac involvement with increased NT-proBNP (789-5716pg/ml, normal range 0-450pg/ml). Patient 4 underwent cardiac MRI which showed interventricular septum and left ventricular wall thickness. In late gadolinium enhancement imaging, myocardium showed delayed enhancement. These findings were consistent with cardiac involvement of amyloidosis. Other five patients who underwent echocardiography showed ventricular wall thickness or interventricular septum thickness with decrease EF (37–46%). One patient had renal involvement with increase creatinine (105μmol/l,normal range 45–84μmol/l).

**Table 1:** Clinical profiles of the patients.

**Case**	**Age/Sex**	**Disease duration before AL Diagnosis (months)**	**Organ Involvement**	**Neurological symptoms**				**Treatment**	**Hematological response**	**Prognosis**
				**CTS**	**Polyneuropathy**	**Autonomic symptoms**	**Pain**			
1	M/56	24	Cardiac involvement	-	+	+	+	NA	NA	Died (3 years after onset)
2	M/57	10	Cardiac involvement	-	+	+	+	MD × 12	CR	
3	M/52	36	Cardiac involvement	-	+	+	+	MD×8	NR	Died (4 years after onset)
4	M/60	7	Cardiac involvement	-	+	+	+	BCD × 5	PR	
5	M/66	6	-	-	+	-	+	BCD × 6	CR	
6	M/64	36	Cardiac involvement	+	-	-	-	RCD × 4	NR	Died (4 years after onset)
7	F/65	36	Cardiac and renal involvement	-	+	+	+	BCD	PR	Died (4 years after onset)
8	M/66	12	-	-	+	+	+	ND	ND	ND

*CTS: carpal tunnel syndrome; NA: not applicable; MD, melphalan + dexamethasone; BCD, bortezomib + dexamethasone + cyclophosphamide; RCD, rituximab + dexamethasone + cyclophosphamide; +, positive; -, negative; CR complete response; PR, partial response; NR, no response; ND, no data*.

The laboratory data are summarized in Table 2. Serum and urine immunofixation revealed positive results in all patients with IgA λ in one, IgG λ in two, λ alone in three, κ alone in one and IgM κ in one. The difference between the involved and uninvolved serum immunoglobulin free light chain (dFLC) levels was elevated in seven patients with available FLC data. The median value was 1,109 mg/L (range, 65–3585 mg/L). CSF protein elevation was present in six cases, with a range of 0.57–1.28 g/L.

**Table 2:** Laboratory data of the patients.

**Case**	**M protein**			**Weight loss (kg)**	**CSF Pro (g/l)**
	**Serum**	**Urine**	**DFLC (mg/L)**		
1	IgA λ	-	NA	30	1.0
2	-	Free λ	65	18	0.88
3	IgG λ	Free λ	294	25	0.85
4	Free λ	Free λ	3,585	10	NA
5	IgG λ	Free λ	564	4	Nor
6	IgM κ	-	2,337	10	1.12
7	-	Free λ	188	20	1.28
8	Free κ	-	731	20	0.57

*d-FLC, difference in free light chain*.

### Electrophysiology

Abnormal electromyography was detected in all patients. Seven Patients showed an abnormal motor conduction velocity (MCV), compound muscle action potential (CMAP), sensory nerve conduction velocity (SCV) and sensory nerve action potential (SNAP), with a more severe amplitude decrease than a prolonged terminal latency and decreased nerve conduction velocity, suggesting severe axonal peripheral neuropathy involving motor and sensory nerves. The changes were more severe in the lower limbs than in the upper limbs in 6/7 cases.

In the bilateral median nerve, one patient showed lower MCV, CMAP, proximal and distal SCV, and SNAP levels, with a prolonged terminal latency. These findings were compatible with the clinical diagnosis of CTS.

Six out of eight patients had abnormal SSR, in which five patients had abnormal SSR in lower limbs and one patient had abnormal SSR in both lower limbs and upper limbs. Two out of eight had normal SSR.

### Pathological Examination

**Figure 1:**
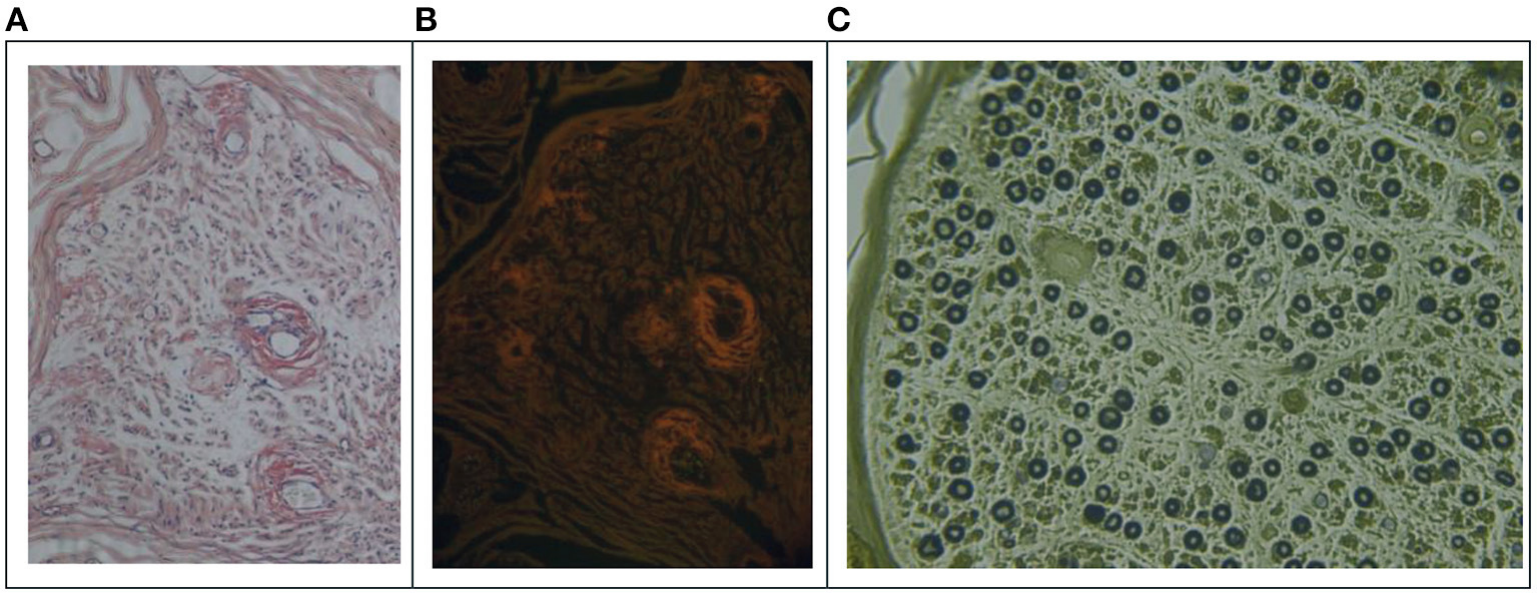
Sural nerve biopsy of the patients. **(A)** Patient 3. Endoneurial perivascular deposition of amyloid (Congo red, original magnification (×200). **(B)** The same section as in A. Amyloid appearing red on immunofluorescence microscopy (×200). **(C)** Patient 2, myelination staining showing myelinated fiber loss, especially small fibers with axonal degeneration and an abnormal vessel wall.

Amyloid material deposits were identified with an irregular thickening of the vessel walls in the endoneurium by sural nerve biopsy in seven patients. At the same time, amyloid deposits were scattered in the endoneurium in six cases (Figure 1). In two patients, amyloid deposits predominated, with an irregular thickening of the vessel walls in the epineurium. The amyloid deposits showed a characteristic apple-green birefringence under polarized light on Congo red staining. Amyloid can be stimulated by immunofluorescence microscopy on Congo red staining (Figure 1).

The main histopathological finding of seven nerve biopsy specimens was nerve fiber loss, with severe and moderate loss in 4/7 and 3/7 cases, respectively, as well as the predominant involvement of small fibers. Active axonal degeneration was noted in three patients, and no regenerative clusters were found in any case.

In the muscle biopsy specimen of case 6, beyond the neurogenic changes, perivascular amyloid material deposition was demonstrated by Congo red staining.

Four patients underwent rectal mucosa biopsy, abdominal wall fat pad biopsy or gingiva biopsy, and amyloid deposition was found in one patient.

### Diagnosis, Treatment and Follow-Up

The median time from onset to diagnosis was 21 months (range 6–36 months). Before the diagnosis of AL amyloidosis was made, two patients were misdiagnosed with CIDP, one patient was misdiagnosed with subacute combined degeneration, and five patients were diagnosed as idiopathic peripheral neuropathy. Two patients underwent months of treatment with steroids and immunoglobulin, and the other patients were treated with multivitamins. In all of the patients, the condition gradually worsened before the anti-plasma cell treatment was administered.

Of the eight patients, the median follow-up time was 10 months (range, 1–14 months). Patient 1 did not receive the anti-plasma cell treatment and died 12 months after being diagnosed with AL amyloidosis. Two patients were treated with MD chemotherapy (cases 2 and 3). Three patients (cases 4, 5, and 7) received BCD (bortezomib + dexamethasone + cyclophosphamide) chemotherapy. One patient (case 6) received RCD (rituximab + dexamethasone + cyclophosphamide) chemotherapy. Hematologically, two patients (cases 2 and 5) achieved a complete response (CR), two (cases 4 and 7) achieved a partial response (PR), and two patients (cases 3 and 6) showed no response (NR). Patients 2 (hematological CR) and 4 (hematological PR) experienced diarrhea relief after 6 and 3 months of chemotherapy, respectively. Patient 5 (hematological CR) experienced significant pain reduction after 3 months of treatment. Patients 3, 6, and 7 died at 10, 12, and 5 months after AL amyloidosis diagnosis, respectively.

## Discussion

In our study, peripheral neuropathy was the initial presenting symptom and the main reason for seeking medical care of all the patients. Before the patients were evaluated at Peking Union Medical College Hospital, all of them had either a nonspecific diagnosis or were misdiagnosed with CIDP or subacute combined degeneration. When peripheral neuropathy appears as the initial symptom of AL amyloidosis, it usually takes a longer time for the correct diagnosis to be made (4). Mathis S. et al. reported five cases of AL amyloidosis mistaken as chronic inflammatory demyelinating polyneuropathy (9). An elevated CSF protein level is one of the reasons why the patients were misdiagnosed with CIDP (9). Fancellu et al. reported a case of AL amyloidosis misdiagnosed as a lower motor neuron disease (10). Tracy et al. reported a patient with multiple upper limb mononeuropathies which nerve biopsy-proven primary amyloidosis (11). All of these serves as a reminder that the recognition of peripheral neuropathy as an early symptom of AL amyloidosis may facilitate making the correct diagnosis at an early stage.

Among our patients, six presented length dependent sensory peripheral neuropathy. Pain was one of the predominant features. Motor involvement typically occurs later in the course of the disease. Nerve conduction studies typically show changes indicating a primarily axonal sensorimotor neuropathy, and needle electromyography shows denervation changes. Adams et al. have also reported similar findings (3). Among our patients, six had prominent autonomic symptoms, which is similar to the findings previously reported in other studies (6). One of our patients presented with bilateral carpal tunnel syndrome and myopathy. Carpal tunnel syndrome is a common manifestation of AL amyloidosis (6). Some patients in other studies also showed lumbosacral radiculopathy and multiple mononeuropathies (3), which serves as a reminder that there are different types of peripheral nerve involvement in AL amyloidosis. Myopathy is also a rare symptom of AL amyloidosis (12).

Seven of our patients were male, and the age at symptom onset ranged from 52 to 66 years. The US Medicare Claims Database shows that AL amyloidosis occurs mainly in male adults over the age of 65 years (13). All patients in our group had significant weight loss and fatigue. In AL amyloidosis, fatigue and weight loss are extremely common presenting symptoms (2, 14). In elderly males with predominant small fiber neuropathy accompanied by pain and autonomic dysfunction, especially those with significant weight loss and fatigue, the diagnosis of AL amyloidosis should be considered.

To make a definite diagnosis of AL amyloidosis, the presence of monoclonal protein is necessary. Among our patients, six had lambda light chains, and two had kappa light chains. In AL amyloidosis, lambda light chains are more common than kappa light chains by approximately 3:1 (2). All seven patients with available FLC data had an abnormal FLC ratio and dFLC level. Therefore, if a patient has neuropathy and a high dFLC level, AL amyloidosis should be considered as a diagnosis and a nerve biopsy may help to confirm the diagnosis.

The diagnosis of AL amyloidosis was confirmed by sural nerve biopsy in seven cases in our study. The nerve pathology shows amyloid deposits in the vessel walls in the endoneurium as well as scattered in the endoneurium. Sural nerve biopsy also showed nerve fiber reduction, axonal degeneration and marked alteration in the fiber size distribution in the sural nerve, with more preservation of large myelinated fibers than of small myelinated and unmyelinated fibers (15). Before undergoing nerve biopsy, four patients underwent lip, abdominal fat pad or rectal biopsy, with negative findings of amyloid deposition in three patients. This suggests that the selection of the biopsy site is still an important issue. Miyazaki et al. recommended that subcutaneous fat aspiration (SFA) and bone marrow biopsies are easy to perform and have a combined sensitivity of 85% for the pathological diagnosis of AL amyloidosis (16). In our experience, if peripheral neuropathy presents as the initial symptom, peripheral nerve biopsy can help to make correct diagnosis. Gillmore et al. also suggested that when possible, a biopsy should be taken from an apparently affected organ to achieve the highest sensitivity and specificity (8).

Patients with AL amyloidosis have a poor prognosis, with an estimated median survival duration ranging from 6 months to 3 years, depending on the patient population and data used (13, 17). In our series, four patients died between 5 and 12 months after diagnosis. Among these four patients, one did not undergo treatment, two had no hematological response, and one achieved a hematological PR. Cardiac involvement and delayed chemotherapy (36 months after onset) are possible reasons for the poor outcomes. In the other three patients who began treatment early (6–10 months after onset), two patients achieved a CR, and one patient achieved a PR. These results indicate that early diagnosis and treatment are critical for managing these patients, and to achieve a better hematological response.

This study highlights the clinical features associated with AL peripheral neuropathy. In cases of elderly patients presenting with predominant sensory peripheral neuropathy associated with severe pain, severe autonomic involvement, weight loss, and fatigue, amyloidosis should be considered. Amyloid deposition found by nerve biopsy or muscle biopsy is very helpful for making a diagnosis. Related M protein examinations can be used to identify the type of amyloid and help to start treatment as soon as possible.

## Data Availability Statement

The original contributions presented in the study are included in the article/Supplementary Material, further inquiries can be directed to the corresponding authors.

## Ethics Statement

The studies involving human participants were reviewed and approved by Ethics Review Committee of Peking Union Medical College Hospital,Chinese Academy of Medical Sciences. The patients/participants provided their written informed consent to participate in this study. Written informed consent was obtained from the individual(s) for the publication of any potentially identifiable images or data included in this article.

## Author Contributions

MQ: original draft, data curation, investigation, and formal analysis. LC and JL: review, editing, and supervision. LQ: review and editing. KS: data curation. HR and YZ: pathology technique. DZ, BP, HG, and YG: supervision. All authors contributed to the article and approved the submitted version.

## Conflict of Interest

The authors declare that the research was conducted in the absence of any commercial or financial relationships that could be construed as a potential conflict of interest.

## Publisher's Note

All claims expressed in this article are solely those of the authors and do not necessarily represent those of their affiliated organizations, or those of the publisher, the editors and the reviewers. Any product that may be evaluated in this article, or claim that may be made by its manufacturer, is not guaranteed or endorsed by the publisher.
