# Supplementary figures and images for: Light-Chain Amyloidosis With Peripheral Neuropathy as an Initial Presentation
Source: Front Neurol. 2021 Sep 28;12:707134. doi: 10.3389/fneur.2021.707134 (PMC8505956; doi:10.3389/fneur.2021.707134)

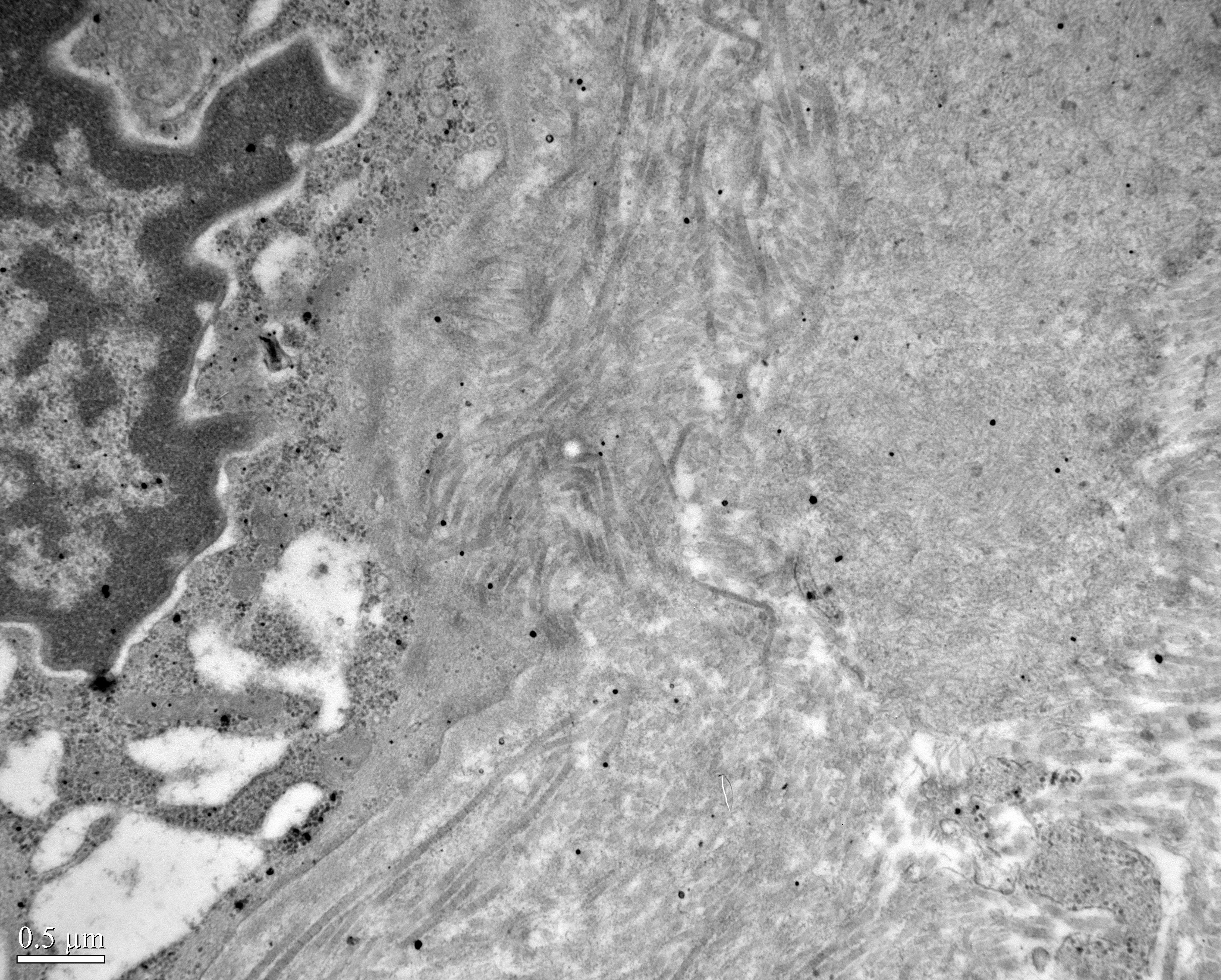

Supplement: Supplementary file 1 [file Image_1.JPEG]
